# Comparative proteomic analysis reveals mechanistic insights into *Pseudomonas putida* F1 growth on benzoate and citrate

**DOI:** 10.1186/2191-0855-3-64

**Published:** 2013-10-25

**Authors:** Manolis Mandalakis, Nicolai Panikov, Shujia Dai, Somak Ray, Barry L Karger

**Affiliations:** 1 Barnett Institute, Northeastern University, Boston, MA 02115, USA; 2 Present Address: Department of Chemistry, University of Crete, Heraklion GR-71409, Greece; 3 Present Address: Harvard School of Public Health, Department of Immunology and Infectious Diseases, Boston, MA 02115, USA

**Keywords:** *Pseudomonas putida*, Benzoate biodegradation, Batch culture, Quantitative proteomics, 2D-LC-MS/MS, Stress response

## Abstract

*Pseudomonas* species are capable to proliferate under diverse environmental conditions and thus have a significant bioremediation potential. To enhance our understanding of their metabolic versatility, this study explores the changes in the proteome and physiology of *Pseudomonas putida* F1 resulting from its growth on benzoate, a moderate toxic compound that can be catabolized, and citrate, a carbon source that is assimilated through central metabolic pathways. A series of repetitive batch cultivations were performed to ensure a complete adaptation of the bacteria to each of these contrasting carbon sources. After several growth cycles, cell growth stabilized at the maximum level and exhibited a reproducible growth profile. The specific growth rates measured for benzoate (1.01 ± 0.11 h^-1^) and citrate (1.11 ± 0.12 h^-1^) were similar, while a higher yield was observed for benzoate (0.6 and 0.3 g cell mass per g of benzoate and citrate, respectively), reflecting the different degrees of carbon reduction in the two substrates. Comparative proteomic analysis revealed an enrichment of several oxygenases/dehydrogenases in benzoate-grown cells, indicative of the higher carbon reduction of benzoate. Moreover, the upregulation of all 14 proteins implicated in benzoate degradation via the catechol ortho-cleavage pathway was observed, while several stress-response proteins were increased to aid cells to cope with benzoate toxicity. Unexpectedly, citrate posed more challenges than benzoate in the maintenance of pH homeostasis, as indicated by the enhancement of the Na^+^/H^+^ antiporter and carbonic anhydrase. The study provides important mechanistic insights into *Pseudomonas* adaptation to varying carbon sources that are of great relevance to bioremediation efforts.

## Introduction

*Pseudomonas putida* is a metabolically versatile Gram-negative bacterium that thrives in habitats as diverse as soils, aquatic systems or the rhizosphere (Timmis 2002). Some *P. putida* strains can utilize a large range of compounds as carbon sources, including aromatic compounds arising from anthropogenic pollution or plant root secretion. In addition, several strains of this bacterium are also selected for a wide range of industrial applications, including biopolymer synthesis (Poblete-Castro et al. 2012). Due to their high biotechnological potential, the genomes of several *P. putida* strains have been fully sequenced (http://img.jgi.doe.gov/cgi-bin/w/main.cgi), but only limited proteomic studies have been conducted to understand the molecular basis of the physiological phenomena that occur under various growth conditions. Since proteins are the functional units of the cell, much can be learned from global proteomic studies (Han et al. 2010).

To date, several proteomic studies of *P. putida* have been conducted to construct lists of identified proteins (protein reference maps) and systematic databases of their functional interactions inside the cell (Cheng et al. 2009a; Park et al. 2009), as well as to investigate the proteomic changes resulting from specific environmental conditions. The latter included the effect on cell growth by toxic heavy metals (Cheng et al. 2009b; Thompson et al. 2010), antibiotics (Yun et al. 2006), synthetic toxic compounds (Loh and Cao 2008; Krayl et al. 2003; Roma-Rodrigues et al. 2010; Santos et al. 2004; Segura et al. 2005; Volkers et al. 2006; Wijte et al. 2011), nutrient deprivation (e.g. iron and nitrogen; Heim et al. 2003; Nikodinovic-Runic et al. 2009) or biofilms formation (Arevalo-Ferro et al. 2005). In several cases, proteomic surveys were also employed to examine the catabolic pathways associated with biodegradation of organic pollutants (Loh and Cao 2008; Cao and Loh 2008; Kim et al. 2006; Verhoef et al. 2010; Yun et al. 2011). Though, beyond catabolic pathways, such studies can provide valuable information about cellular physiology and the adaptation mechanisms activated under the presence of the pollutant. Ultimately such information can be utilized to genetically engineer microorganisms with improved properties (e.g. efficient removal of toxic wastes) (Han et al. 2011).

The majority of published proteomic studies to date have employed conventional two-dimensional polyacrylamide gel electrophoresis (2D-PAGE), followed by mass spectrometry. 2D-PAGE is, however, being replaced today by LC-MS/MS shotgun proteomic methods due to the automated approach and the larger number of proteins identified and quantitated. Up to now, only two proteomic studies on the degradation of monoaromatic compounds by *P. putida* strains have employed shotgun proteomics using two dimensional liquid chromatography and offline analysis of peptides by tandem mass spectrometry (offline 2D LC-MALDI-TOF-MS/MS) (Kim et al. 2006; Yun et al. 2011). Both of these studies employed stable isotope labeling approaches for protein quantitation, but the largest number of proteins identified and quantitated was only 570.

While *P. putida* F1 is a fully sequenced strain, widely used in bioremediation applications (Díaz et al. 2008; Friman et al. 2012; Parales et al. 2000), only a handful of studies have investigated its catabolic versatility using proteomic approaches. In the present work, we explored the effect of two contrasting carbon sources on growth kinetics and the accompanying changes in the proteome of *P. putida* F1. Citrate, a carbon source that is readily assimilated through central metabolic pathways, and benzoate, a monoaromatic compound of medium toxicity that is degraded via peripheral catabolic pathways, were chosen as carbon substrates. This selection was also based on the fact that benzoate is frequently used as a model compound for oil-related monoaromatic pollutants in the environment (Carmona et al. 2009), while citric acid is ubiquitous in nature as a key cellular intermediate.

Using Tandem Mass Tag (TMT) isobaric labeling of peptides in conjunction with 2D LC-ESI-MS/MS analysis on an LTQ Orbitrap XL mass spectrometer, an in-depth proteomic profiling of *P. putida* F1 was performed, and key proteins involved in benzoate and citrate biodegradation were detected. Importantly, several other functional groups of proteins were preferentially expressed on one or the other of the primary carbon sources. These proteins were related with a variety of cell features and adaptation processes, the exact role of which was thoroughly investigated. Changes in the available carbon sources are very frequent in natural ecosystems, and the present proteomic study provides insight into the biological processes of *P. putida* F1 that can be affected by a change of the carbon source between distinctly different organic substrates.

## Material and methods

### Bacteria strain and growth conditions

The *Pseudomonas putida* F1 strain (ATCC 700007) was kindly provided by Dr. Rebecca E. Parales (UC Davis, CA). Primary cultures and all cultivation experiments in this study were established in liquid media consisting of a modified Hutner’s Mineral Base (HMB) (Reardon and Kim 2002) supplemented with trisodium citrate or sodium benzoate as the sole carbon-energy source. The temperature was maintained at 33°C, which is optimum for *P. putida* growth (Alagappan and Cowan 2004), while pH was kept constant at 6.6.

A BIOFLO 2000 fermentor (New Brunswick Scientific Co., New Brunswick, NJ), consisting of a 2-liter glass vessel, was used to grow the bacteria under computer-aided control of temperature, pH, pO_2_ and stirring rate. The system was aerated with a constant airflow adjusted at 400 mL min^-1^ using an electronic mass flow controller (FMA 1700, OMEGA Engineering Inc., Stamford, CT). The stirring rate was set at 1000 rpm to maintain a dissolved oxygen concentration above 50% of air saturation. An infrared analyzer LI-800 (LI-COR Biosciences, Lincoln, NE) was connected to the air outlet for continuous monitoring of CO_2_.

To culture bacteria, the fermentation vessel was filled with 1.5 L of HMB medium with 1.0 g/L of benzoate or citrate and then inoculated with 20 mL of an overnight shake flask culture. Culture development was monitored as respiration rates of CO_2_ evolution and O_2_ uptake, as well as by measuring optical density at 600 nm (OD600) of aliquots collected at 10–60 min intervals with a refrigerated BioFrac fraction collector (Bio-Rad Laboratories, Hercules, CA). Total organic carbon content of cells was derived from OD600 measurements based on a preliminary calibration: cell suspensions of *P. putida* with varying OD600 were centrifuged, and cell pellets were washed, dried, ignited to CO_2_ (Teledyne Tekmar 183 Boat Sampler) and quantified with a UGA200 mass spectrometer (Stanford Research Systems, CA).

### Analysis of substrates and metabolites

Culture supernatants were analyzed by a Shimadzu VP series HPLC-UV system (Shimadzu Corporation, Kyoto, Japan) using a ZORBAX Eclipse Plus C18 column (30 × 4.6 mm, 3.5 μm; Agilent Technologies, Andover, MA). The UV-detector was operated at 275 nm and 200 nm for the detection of benzoate and citrate, respectively. A gradient of acetonitrile in 0.1% formic acid (8% acetonitrile for 2 min, linear increase to 35% in 10 min) was applied for the analysis of benzoate and its metabolites, while isocratic elution with 0.01 N H_2_SO_4_ was used for citrate.

Metabolites of benzoate were identified by gas chromatography–mass spectrometry (GC-MS). An aliquot (1 mL) of cell culture was centrifuged, and the supernatant was evaporated to dryness in a SpeedVac SC110A concentrator (Savant Instruments, Holbrook, NY). The solid residue was derivatized with N-methyl-N-(tert-butyldimethylsilyl) trifluoroacetamide, centrifuged, and the supernatant analyzed on a GC-MS system (Agilent 6890 GC with 5973 mass-selective detector; Agilent Technologies, Palo Alto, CA). Metabolites were identified by comparison of MS spectra with the NIST 08 mass spectral library.

### Comparative proteomic analysis

Approximately 50 mg of *P. putida* cells were harvested during the exponential phase of benzoate and citrate batch cultures, washed twice with sterile phosphate-buffered saline (pH 7.4) and resuspended in 150 μL lysis buffer (7 M urea, 2 M thiourea, 4% CHAPS, 25 mM HEPES pH 7.5, 30 mM dithiothreitol, 1 mM EDTA) containing 1X Halt Protease Inhibitor Cocktail (Thermo Fisher Scientific, Pittsburgh, PA). The mixture was sonicated on ice (eight 10 s pulses with 20 s cooling intervals) by using a 1/8-inch sonication probe (FB-50, Thermo Fisher Scientific). Any unbroken cells and debris were removed by centrifugation, and the total protein concentration from the supernatant was determined using the BCA assay (Microplate BCA Protein Assay Kit; Thermo Fisher Scientific).

Protein reduction, alkylation, trypsin digestion and labeling of the resulting peptides were accomplished using a tandem mass tag (TMT) isobaric labeling kit (Thermo Fisher Scientific, San Jose, CA) according to the manufacturer's instructions. Briefly, each lysate (100 μg protein) was diluted in 90 mM tetraethyl ammonium bicarbonate, reduced with 10 mM tris-(2-carboxyethyl) phosphine and alkylated with 18 mM iodoacetamide. Proteins were then digested overnight with 2.5 μg of trypsin (trypsin/protein ratio of 1:40) at 37°C (pH 8), and the peptides corresponding to benzoate- and citrate grown cells were isobarically labeled with TMT reagents. The reaction was quenched with 5% hydroxylamine, and equal amounts of differentially TMT-labeled peptides were then pooled together.

The pooled sample was fractionated by high-pH reverse phase HPLC, as previously described (Dowell et al. 2008). Briefly, the pooled peptide mixture was evaporated to dryness and reconstituted in 20 mM ammonium formate buffer (pH 10, mobile phase A). The peptides were loaded on a Zorbax 300 Extend-C18 column (2.1 × 150 mm, 3.5 μm; Agilent Technologies) at a flow rate of 200 μL min^-1^ and separated using a linear gradient from 2% to 42% of mobile phase B (90% acetonitrile in 20 mM ammonium formate buffer) over 35 min. The elution profile was monitored at 214 nm with fractions being collected every 1.5 min. A total of 20 fractions were further subjected to nanoLC MS/MS analysis.

#### NanoLC MS/MS analysis

Fractionated peptides were separated in a second dimension of low pH reversed phase chromatography coupled to mass spectrometry. The platform consisted of an Ultimate 3000 nano-LC pump (Thermo Fisher Scientific), a self-packed capillary column (75 μm i.d. × 20 cm length) with Magic C18AQ particles (3 μm i.d., 200 Å pore size; Michrom BioResources, Auburn, CA), an LTQ-Orbitrap XL mass spectrometer (Thermo Fisher Scientific) equipped with a PicoView nanospray interface (New Objective, Woburn, MA). Each fraction was evaporated to dryness, reconstituted in 10 μL of 0.1% formic acid in water (mobile phase A), and 1 μL was loaded onto the column at a flow rate of 250 nL min^-1^. After desalting for 30 min with 2% mobile phase B (0.1% formic acid in acetonitrile), the peptides were eluted at 200 nL min^-1^ with a linear gradient of B: 2% to 5% in 2 min, 5% to 35% in 240 min, and 35% to 90% in 10 min.

The mass spectrometer was operated in the positive ionization mode with data acquisition in ~3.5 s per scan cycle, starting with a full MS scan of the precursor ions in the Orbitrap (400 to 1600 m/z; resolution of 30,000 at m/z of 400; automatic gain control (AGC) of 5 × 10^5^), followed by three data-dependent CID MS/MS scans of the three most abundant precursor ions in the ion trap and then three HCD MS/MS scans on the same precursor ions in the Orbitrap (resolution of 7500). The normalized activation energy of CID and HCD were set at 35% and 48%, and the AGC at 5 × 10^3^ and 1 × 10^5^, respectively. For both scan modes, the minimum MS signal for triggering MS/MS was set at 1,000, while dynamic exclusion was implemented with a repeat count of 1 and exclusion duration of 40 s. Details about the computational analysis of shotgun proteomics data, including data filtering, peptide/protein identification and quantification, statistical analysis of quantitative data, as well as the functional categorization of the differentially expressed proteins are provided in the Additional file 1.

## Results

### Growth dynamics in single and repetitive batch cultures

**Figure 1:**
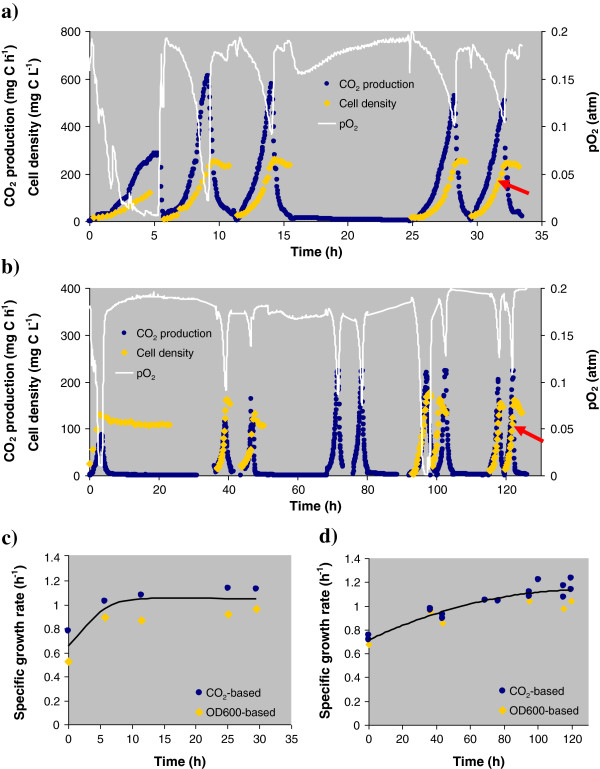
**Repetitive batch growth of *P. putida *F1 in a bioreactor using a) benzoate or b) citrate as the sole carbon and energy source (cell growth was followed by measuring optical density at 600 nm, CO**_**2 **_**production and O**_**2 **_**consumption).** Arrow indicates the sampling time for proteomic analyses. The specific growth rates derived from the exponential phase for each growth cycle in **c)** benzoate or **d)** citrate are also shown.

To avoid the artifacts and uncertainties associated with an incomplete adaptation of the bacteria to carbon/energy sources, we ran batch cultures in a repetitive mode for at least 5 cycles. In each cycle, cells were allowed to deplete the limiting nutrient, and then 90% of the cell suspension was replaced with fresh media to initiate a new cycle. Figure 1 shows a long-term plot of repetitive batch growth versus time for both substrates. After each addition of fresh media, we observed a steep increase in cell mass accompanied by a synchronous rise of respiratory activity (increase of CO_2_ and decrease of dissolved O_2_). Initially (1 to 3 growth cycles), bacterial dynamics was found to be irregular with somewhat slower growth and lower cell yields. Then, the growth pattern became stable and reproducible (Figures 1a and 1b), with minimal lag-phase and a maximum SGR merging to 1.0 h^-1^ (Figures 1c and 1d).

**Figure 2:**
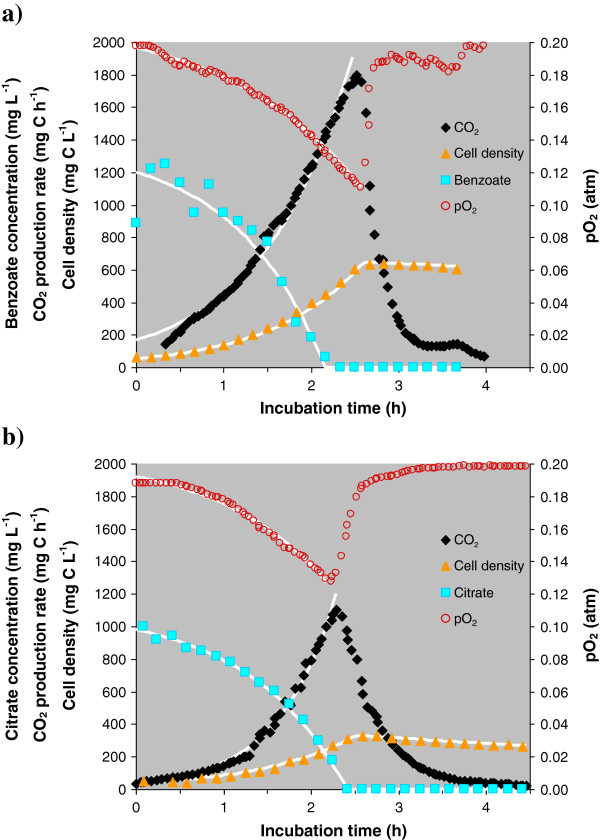
**Growth profile of *P. putida *F1 in a) benzoate or b) citrate-containing media after bacterial activation by repetitive growth cycles.** Markers represent experimental data points for cell biomass (triangles), CO_2_ production rate (diamonds), pO_2_ (circles) and residual substrate (squares), while the white lines correspond to model results for the respective parameters (Additional file 1: Table S3).

Figure 2 shows a single growth cycle of the stabilized and activated bacterial culture in each substrate after 5 repetitive cycles. Immediately after addition of fresh medium, the bacterial cell mass began an exponential increase accompanied by a synchronous exponential rise in respiration rate and C-substrate uptake. After 2 to 3 h, the exponential phase was abruptly terminated by the C-source depletion. Cell samples were collected for proteomic analysis during the exponential phase (cell density at 75% of maximum OD600) of the final growth cycle, as indicated by the arrows in Figure 1.

### Proteomic results

In total, 514,914 tandem mass spectra were acquired from the 2D LC-MS/MS analysis of the TMT-labeled peptide mixture, with 99,379 being matched to peptides (at an FDR < 1%) after MASCOT database searching. From these data, a list of 26,678 unique peptides was derived and assigned to 2,606 proteins, of which 2,141 were identified with at least two unique peptides (41% coverage of *P. putida* F1 proteome). Moreover, roughly 1,900 of these proteins were quantitated with at least 2 unique peptides.

**Figure 3:**
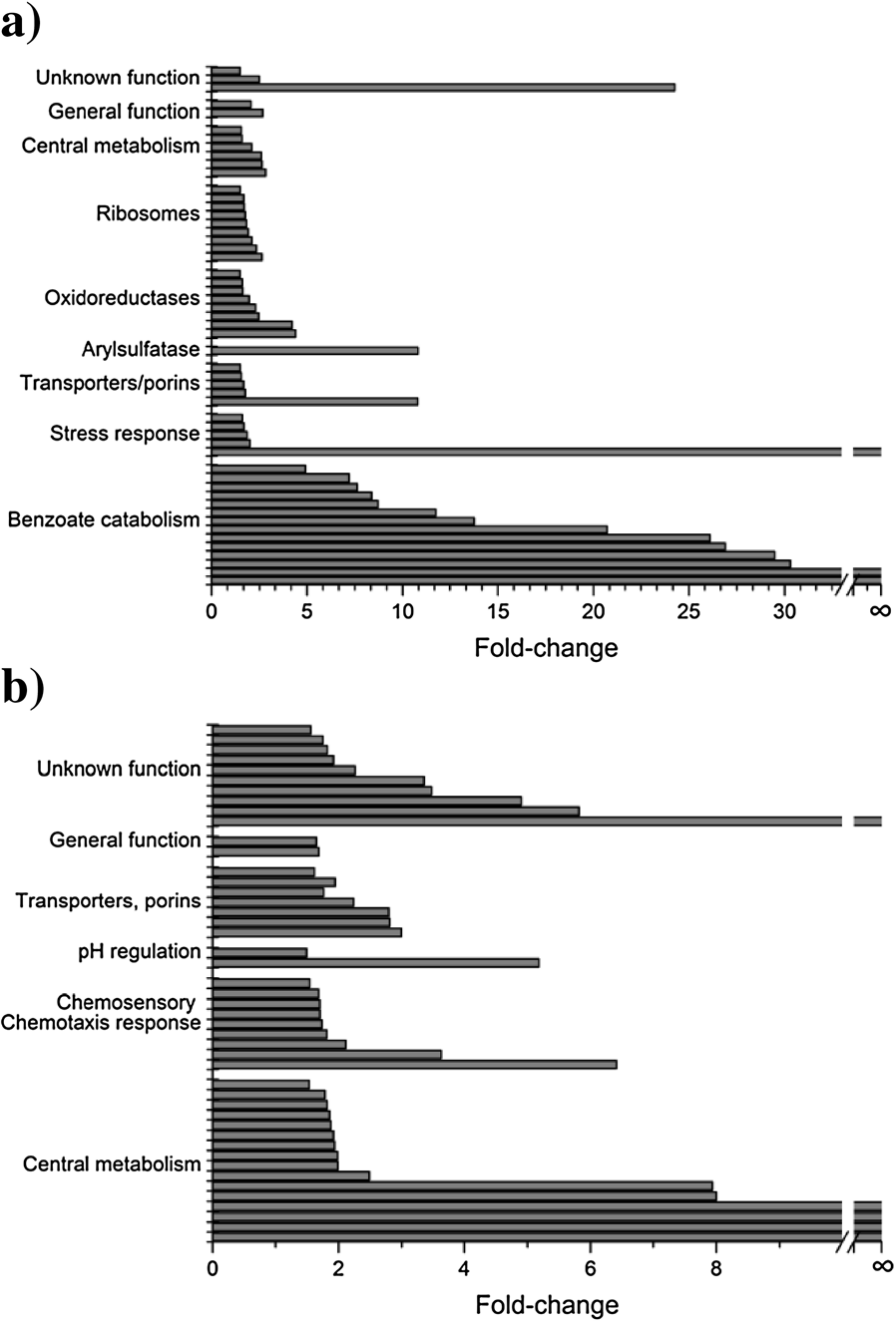
**Fold-change of the functional groups that were preferentially expressed in *P. putida *** **F1 growth on a) benzoate and b) citrate.**

To visualize the results from the comparative proteomic analysis and to pinpoint differentially expressed proteins, a ‘volcano plot’ depicting log_2_ fold-change of proteins against statistical significance (negative log_10_ of p-value) was generated (Additional file 1: Figure S1). This analysis revealed 53 upregulated proteins under benzoate growth conditions, with three of the proteins being found only in the benzoate-grown cells (Additional file 1: Table S1). Citrate-grown cells showed 46 upregulated proteins, five of which were found only in the citrate-grown cells (Additional file 1: Table S2). All these proteins were clustered into functional groups (Figure 3) indicating particular metabolic processes responsible for the utilization of the different carbon sources and the physiological adaptation of *P. putida* cells to them. Catabolic enzymes were upregulated in benzoate (relative to citrate) as well as other proteins responsible for regulation of redox balancing, ribosomal synthesis, and stress-resistance. On the other hand, proteins related to chemosensory functions and ion balance/pH homeostasis were more expressed in cells cultivated on citrate. In addition, several proteins involved in central metabolism and transmembrane transportation were identified in both substrates, as well as a substantial number of proteins with unknown function.

### Extracellular intermediates of benzoate degradation

The culture supernatants analyzed by HPLC-UV over the course of benzoate degradation revealed the formation of two metabolites (Additional file 1: Figure S2), which were tentatively identified as catechol and *cis,cis*-muconate based on retention time, in agreement with published data (Li et al. 2010). By the GC-MS analysis of an additional aliquot from the exponential growth phase, we were able to confirm the presence of catechol, as well as of oxoadipate and succinate intermediates, but it was not possible to detect *cis,cis*-muconate. The inability to detect the latter metabolite and other benzoate metabolites could be explained by the ephemeral nature and/or the incomplete coverage of these compounds (i.e. their silylated derivatives) in the mass spectral library used for GC-MS identification.

## Discussion

The results of the microbiological parameters monitored over the course of benzoate and citrate batch cultures were quantitatively analyzed to identify which changes in *P. putida* F1 growth characteristics could be explained by the different properties of the carbon substrates. In the following, the growth kinetics and bacterial physiology are first discussed, followed by a comprehensive examination of the proteome results.

### Growth kinetics and stoichiometry

The growth pattern of a single batch cycle in activated *P. putida* F1 (Figure 2) was much simpler than a typical pattern of bacterial growth, as two distinctive phases (lag-phase and decelerated growth phase) were practically not observed. The exponential and stationary phases were successfully fit to a simplified version of the Monod model (Pirt 1975), which assumes an abrupt transition from unlimited exponential growth to a non-growing state (Additional file 1: Table S3). The calculated curves from this model, added to Figure 2, demonstrate fairly good agreement with experimental data points. The lack of a lag-phase is self-evident because repetitive batch cultivation removes any damaged, dormant or abnormal cells during earlier cycles, and, at time zero, all cells in the bacterial population are active and ready for growth. The lack of a deceleration phase indicates that the bacterial specific growth rate (SGR) is not significantly affected by substrate concentration until it is completely depleted. This result is reasonable if the transporters of carbon substrates have a very low saturation constant (i.e. the transport processes provide efficient uptake of the substrate even at very low concentrations) and if there is no product inhibition (Pirt 1975; Panikov 2011).

Two conclusions follow from the kinetic analysis. The first is that the SGR can be measured within the exponential growth phase not only as a plot of OD600 *vs* time, but also as a plot of respiration rate or substrate uptake rate *vs* time. Figures 1 and 2 provide clear evidence that both SGR-estimates based on OD600 and CO_2_ production agree with each other. The rates of CO_2_ production and oxygen uptake are particularly convenient measures of growth as they can be determined automatically and with higher accuracy than OD600.

The second conclusion is a precautionary note regarding the selection of cellular material for proteomic analyses. Up to now, insufficient attention has been paid to the stabilization of the growth profile, and the collection of exponentially grown cells in previous studies was generally conducted without prior repetitive batch cultivation. Importantly, the actual growth intensity observed in ad hoc batch cultivation without preliminary sequential growth passages can be significantly lower than the ‘correct’ SGR attributed to a given bacterial strain. For example, the first batch cycle on benzoate and citrate (Figure 1c, 1d) yielded an SGR that was 30 to 35% lower than the maximum SGR. The reduced growth rate could be accompanied by cellular heterogeneity (i.e., some cells being more active than others) and thus with an altered proteome profile as compared with the fully activated culture.

On average, the SGR was equal for benzoate and citrate (1.01 ± 0.11 and 1.11 ± 0.12 h^-1^, respectively). On the other hand, the cell yield was consistently higher on benzoate (0.6 g cell/g substrate) than on citrate (0.29 g/g), as expected, based on thermodynamic considerations (Esener et al. 1983; Panikov 1999; Roels 2009), i.e., 1 g/L each of trisodium citrate and sodium benzoate require, respectively, 17 mM and 52 mM O_2_ for their complete oxidation. These results indicate that benzoate contains 3-fold more energy per unit mass than citrate. Below we show that these thermodynamic considerations are consistent with proteomic differences.

### Preferentially expressed proteins in benzoate-grown *P. putida* F1

#### Enzymes of the benzoate catabolic pathway

**Figure 4:**
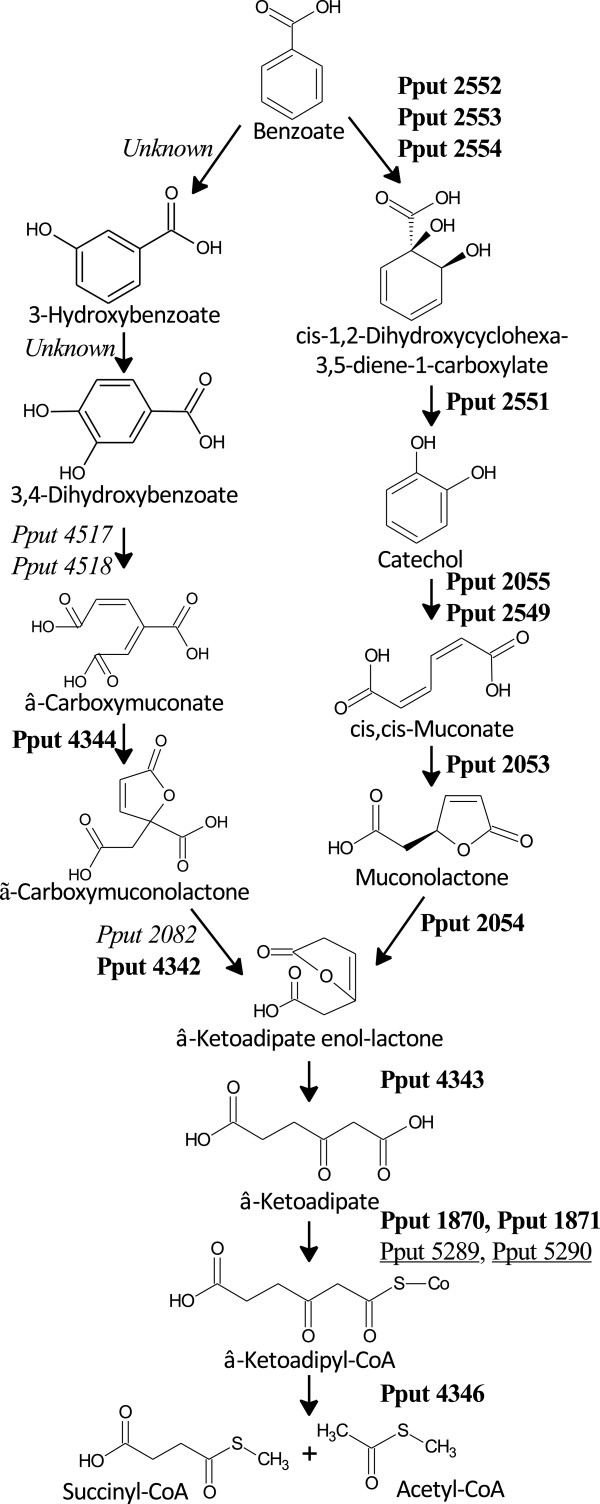
**KEGG pathway map for benzoate degradation in *P. putida *F1.** The proteins identified as upregulated by one (underlined) or at least two peptides (in bold) are highlighted. Undetected proteins are also shown in italics.

Out of the 54 upregulated proteins found in benzoate-grown cells, not surprisingly, 14 were associated with the catabolic degradation of benzoate (Harwood and Parales 1996). More specifically, 12 proteins covered all eight steps of benzoate degradation via the catechol ortho-cleavage pathway (Figure 4), including the initial oxidation of benzoate to a 1,2-dihydroxy derivative and the eventual formation of succinyl-CoA and acetyl-CoA intermediates of the TCA cycle. It should be noted that two additional enzymes participating in the transformation of *β*-ketoadipate to *β*-ketoadipyl-CoA (Pput_5289, Pput_5290), while being quantified by only one peptide each, nevertheless showed a strong upregulation (6- to 11-fold). By including the latter two proteins, we identified the complete list of proteins responsible for benzoate degradation via the catechol ortho-cleavage pathway, as predicted from the full genome of *P. putida* F1. Additional evidence for the importance of the specific degradation process was provided by the analysis of metabolites in the culture supernatants. Catechol, *cis*,*cis*-muconate, oxoadipate and succinate, identified by either HPLC-UV or GC-MS measurements, were all benzoate metabolites arising from the catechol ortho-cleavage pathway.

Another two significantly upregulated proteins (Pput_4344, Pput_4342 with 8- to 27-fold change, respectively) were found to be involved in the protocatechuate ortho-cleavage, which is a parallel branch to the catechol ortho-cleavage pathway (Harwood and Parales 1996). This branch mediates the transformation of hydroxybenzoate to *β*-ketoadipate enol-lactone. The latter metabolite is a common intermediate of both the catechol and protocatechuate branches, and it thus represents the point of metabolic convergence (Figure 4). The two upregulated proteins (Pput_4344, Pput_4342) were found to catalyze the transformation of *β*-carboxymuconate to γ-carboxymuconolactone and then to *β*-ketoadipate enol-lactone. While the change in the level of these enzymes was previously observed in p-hydroxybenzoate and vanillin biodegradation (Kim et al. 2006), this is the first report demonstrating that such a change can occur during the degradation of benzoate by *P. putida* F1. Our finding suggests that a fraction of benzoate could initially be hydroxylated and then channeled to the protocatechuate branch. However, the additional enzymes required for the transformation of benzoate to protocatechuate and then to β-carboxymuconate were not detected in this study, possibly due to their relatively lower abundances compared with the observed enzymes. Nevertheless, additional experiments (e.g. gene-knockdown experiments) will be needed to clarify the precise biological importance of Pput_4344 and Pput_4342 on benzoate degradation.

The above results confirm the strong biodegradation potential of *P. putida* F1 against monoaromatic compounds and indicate that this specific bacterium can lead to the complete degradation of benzoate. This result is of major importance for bioremediation-related applications where efficient cleanup of contaminated environments is required.

#### Stress-response proteins

As compared to citrate, cell growth on benzoate resulted in as much as a 2-fold increase in the level of several stress-related proteins. Included in this group were GroEL chaperonin (Pput_4363) and an Hsp70 chaperone DnaK (Pput 4593), both of which promote the proper folding of proteins under stress conditions (e.g. heat-shock, chemical stress), as well as an hslU protease (Pput_4875) participating in the degradation of misfolded proteins (Rosen et al. 2002). Another enzyme, an ATP dependent helicase of the Lhr-family (Pput_1102), was only observed in benzoate-grown cells. Lhr-protein (i.e. long helicase-related) is the largest known protein in *Escherichia coli*, containing all seven conserved motifs of the DNA and RNA helicase superfamily II (Reuven et al. 1995). The Lhr gene has been reported to play a critical role in stimulating DNA repair in *Caulobacter crescentus* (Martins-Pinheiro et al. 2007) and *Mycobacterium tuberculosis* (Guo et al. 2010). Benzoate is known for its mutagenic ability, as shown in the case of yeast mitochondrial DNA (Piper 1999). Thus, a likely function of the Lhr protein in *P. putida* cells is the repair of benzoate-induced DNA damage.

### Transporters

One putative uncharacterized protein (Pput_4340), upregulated by 11-fold in benzoate-grown cells, was 100% identical with an outer membrane *benF*-like porin of *P. putida* KT2440. In *P. putida*, *benF* is part of an operon involved in benzoate catabolism regulated by *benR* (Cowles et al. 2000; Nishikawa et al. 2008; Sampathkumar et al. 2010). Although the function of the porin is not completely known, there is some evidence, based on the heterological expression of the respective gene in yeast, that the *benF* gene product functions as an efflux pump of benzoate (Nishikawa et al. 2008). Thus, the likely physiological role of the protein in *P. putida* F1 is the relief of cells from benzoate toxicity by pumping this substrate out of the cells.

A sulfate transporter (Pput_1565), along with three proteins involved in the transport of low-molecular-weight nitrogen-containing compounds, such as cystine/diaminopimelate (Pput_0242), glutamate/aspartate (Pput_1112) and glycine/betaine (Pput_0316), were upregulated up to 1.8-fold. Nitrogen-containing compounds of low molecular weight, and glycine/betaine, in particular, have been suggested to play an osmoprotective role in several types of bacteria (Komarova et al. 2002). Moreover, the upregulation of amino acid and sulfate transporters has been previously observed in *P. putida* KT2440 under tetracycline-induced chemical stress (Yun et al. 2006). Considering that the osmolarity of the benzoate- and citrate-containing culture media used in the present study was not substantially different, it can be inferred that the upregulation of the specific transporter proteins in benzoate-grown *P. putida* F1 was more likely the result of a higher chemical rather than osmotic stress induced by benzoate.

### Sulfatase

A sulfatase (Pput_2406) that was upregulated by 11-fold in benzoate-grown cells, was 96% identical with an arylsulfatase (PP_3352) annotated in *P. putida* KT2440. Sulfatases, i.e. enzymes that catalyze the hydrolysis of sulfate ester bonds in a variety of organic sulfates, play an important role in the survival of *P. putida* in the soil environment (Kertesz 2000; Kahnert et al. 2002; Mirleau et al. 2005). This is quite reasonable, considering that sulfur in soils is mainly in an organic form, represented by sulfonates, sulfate esters, as well as sulfur-containing amino acids (methionine, cysteine and their sulfoxide and sulfone derivatives).

On the other hand, the presence of inorganic sulfate is known to decrease the expression of several bacterial sulfatases (Kertesz 2000). While sulfate was an ingredient of the HMB medium used in both benzoate and citrate cultures, comparative proteomic analysis indicated a significant preferential expression (over 11-fold) of arylsulfatase (Pput_2406) in benzoate-grown cells. Further studies are needed to explore whether the presence of aromatic (e.g. benzoate) rather than aliphatic (e.g. citrate) C-substrate is able to override the repressive action of inorganic sulfate. From an ecological perspective, such metabolic regulation would be advantageous for the survival of *P. putida*. If naturally occurring aromatic hydrocarbons are at least partly sulfated, then arylsulfatase repression by coexisting inorganic sulfates would be an obstacle to the consumption of available sources of organic carbon, which is generally the limiting factor for bacteria growth in real ecosystems.

### Other catabolic enzymes, tentative redox-balancing function

Several oxidoreductases, mainly represented by dehydrogenases, oxygenases and other redox-balancing proteins were preferentially expressed in benzoate-grown cells (Additional file 1: Table S1). It is tempting to speculate that upregulation of oxidoreductases is required for the balancing of the redox state of cells when grown on a highly reduced C-substrate such as benzoate. The difference in the degree of reduction was identified through a comparison of the elemental composition of benzoate, citrate and of *P. putida* F1 cell mass by using brutto-formula normalized to C-content (Table 1). The average oxygen content in cells is 0.5 atoms per carbon, which is substantially higher compared to benzoate (0.29) and much lower than that of citrate (1.16). With this in mind, enzymes catalyzing oxygen incorporation into carbon substrates would be relatively more advantageous for cells growing in benzoate than in citrate. The benzoate catabolic pathway (Figure 4) involves several oxidative steps bringing the end products (succinate and acetate) close to the cell-mass average level. However, some intermediates are likely to be diverted from the catabolic path to anabolic reactions, such as the synthesis of aromatic amino acids and nucleotides *etc*. Consequently, additional oxygenases (i.e. other than those enzymes involved in the benzoate ortho-cleavage pathway) would be required by the cells in order to compensate for the oxygen deficiency. The results from our proteomic analysis appear to be consistent with this reasoning.

**Table 1:** Brutto-formula of benzoate, citrate and cell mass normalized to carbon content

**Benzoate**	**Citrate**	**Average cell**^**a**^
CH_0.71_O_0.29_Na_0.14_	CH_0.83_O_1.16_Na_0.5_	CH_1.9_O_0.5_ M_0.2_

^a^The M symbol represents bulk mineral instead of Na alone.

### Ribosomes

A group of 9 ribosomal proteins were upregulated under benzoate-growth conditions. The synthesis of ribosomal proteins is tightly coupled to cell growth rate, and high levels of ribosomes are a common feature of fast growing cells. In this context, the preferential expression of ribosomal proteins in benzoate rather than in citrate is not very clear, considering that *P. putida* F1 cells exhibited the same specific growth rate in both substrates.

Though, this effect may be indicative of a stress response arising from cells exposure to high benzoate levels. In a previous transcriptomic study, Jang et al. (2008) investigated the cellular response of *Staphylococcus aureus* to phenylphenol and they observed a strong overexpression of several genes encoding ribosomal proteins after 20 min of exposure. This finding was interpreted to reflect a bacterial response to phenylphenol toxicity and it was argued that the upregulation of ribosomal protein genes might enhance the translation process or help proper ribosome functioning under stress conditions as the exposure to phenylphenol. Based on these, a similar response would be expected for *P. putida* F1 exposure to benzoate considering its moderate toxicity and its structural similarities to phenylphenol.

### Preferentially expressed proteins in citrate-grown *P. putida* F1

#### Central metabolism

A total of 16 proteins involved in central metabolism (Figure 3b) were upregulated (relative to benzoate) in citrate-grown cells. These proteins were distributed among key metabolic processes, such as citrate cycle, energy transduction, biosynthesis of some cellular components (e.g. fatty acids, thiamine) and catabolism of amino acids (i.e. glycine, aspartate) (Additional file 1: Table S2). The specific changes most probably occurred as a manifestation of the entire metabolic network change in switching from one C-source to another.

#### Transporters

The complete genome of *P. putida* F1 lists five proteins attributed to citrate transport; however, only one of them, Pput_0165, was detected and quantified (1 peptide) with a high citrate-induced upregulation (>15-fold), suggesting that citrate/proton symport can be a main citrate entry into the cell. In contrast to benzoate growth conditions where transporters for small nitrogen-containing molecules were upregulated, the group of citrate-induced transporters was primarily composed of outer membrane porins. A total of four outer membrane porins (Pput_1235, Pput_0249, Pput_1128, Pput_4302), as well as a TonB-dependent siderophore receptor (Pput_0283), the physiological function of which could be counter-balancing the intracellular Fe chelating by citrate, were upregulated. Additionally, two upregulated proteins in citrate-grown cells were responsible for protein translocation: preprotein translocase subunits SecE (Pput_0474) and YajC (Pput_0864). Their physiological/functional roles as well as the reason to be stimulated by citrate need further study.

### Ion balance and pH regulation

**Figure 5:**
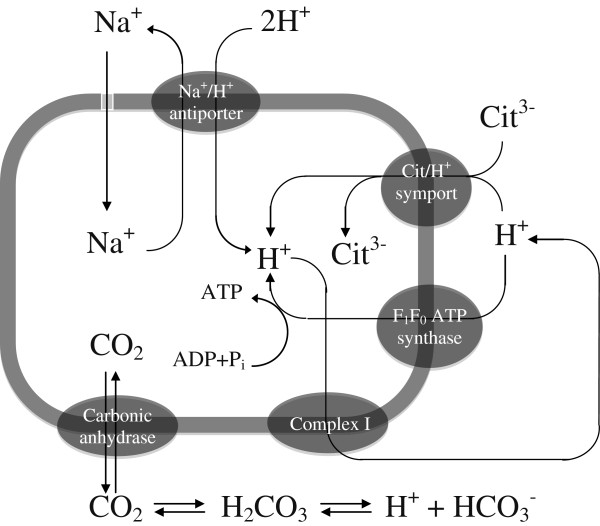
**Hypothetical mechanisms of metabolic alkalinity neutralization.** Proteins involved: **i)** NhaA, Na^+^/H^+^ antiporter, **ii)** Citrate/H^+^ symport, **iii)** F_1_F_0_-ATP synthase, **iv)** Complex I: NADH–ubiquinone oxidoreductase and **v)** Carbonic anhydrase/carbonate dehydratase.

Na^+^/H^+^ antiporter Nha (Pput_1868) increased its level in citrate-grown cells, relative to benzoate, by a factor of 5.2. The major reason for the increase is likely to be the process of alkalization caused by excess of sodium (Sakano et al. 1997). The difference between sodium citrate and sodium benzoate is that the former generates three uncompensated Na^+^ ions per consumed molecule versus one Na^+^/molecule for benzoate. Therefore, the cell faces a higher demand to maintain the cytoplasm near neutral pH while growing on citrate. The scheme in Figure 5 summarizes a postulated mechanism of neutralizing Na^+^ by using three metabolic mechanisms: i) Na^+^/H^+^ antiporter Nha pumping Na^+^ out of the cell, ii) carbonate dehydratase (Pput_0115 ) that catalyzes rapid interconversion of CO_2_ (respiration products) and water to bicarbonate and protons to compensate for the excessive alkalinity of culture medium, and iii) catabolic breakdown of intracellular amino acids (e.g. glycine, aspartic acid; based on the upregulation of Pput_1026, Pput_5247) (Sakano et al. 1997), which produce weaker ammonium cations and relatively stronger organic acids. The cumulative effect of the three metabolic mechanisms makes it possible to maintain a neutral medium. In our experiments, the amount of 1 N HCl used to neutralize metabolic alkalinity was roughly the same in the citrate and benzoate fermentations, probably due to the above neutralization mechanisms in citrate-grown cells.

### Chemosensory/chemotaxis proteins

Citrate-grown cells showed a preferential expression of several proteins involved in flagellar motility (e.g. flagellar basal body-associated protein FliL, Pput_1507) and cell chemotaxis, e.g. CheW chemotaxis adaptor proteins, methyl-accepting chemotaxis proteins (MCPs) and chemotaxis histidine kinases (Additional file 1: Table S2). The mechanism of bacterial chemotaxis is primarily controlled by a core signaling system composed of membrane chemo-receptors (i.e. MCPs), cytoplasmic histidine kinases (widely known as CheA proteins) and CheW proteins which facilitate the coupling between them (Liu et al. 2012). This system senses chemical signals and transduces them to the flagellar motor to control the direction of rotation (Liu et al. 2012). The increase of MCPs, histidine kinase and CheW proteins observed in *P. putida* F1 suggests that chemotactic activity was higher in citrate than in benzoate-grown cells. Most probably, this comparative result is another side effect driven by the moderate toxicity of benzoate rather than a direct positive effect from citrate. In fact, a previous transcriptomic study investigating toluene stress in *P. putida* KT2440 reported a repression of the motility/chemotaxis and pilus/flagellum-related genes as a result of aromatic toxicity (Domínguez-Cuevas et al. 2006). It was further suggested that since motility is a highly energy-consuming function, the repression of motility genes may serve as an adaptive response of bacteria to save energy in coping with chemical stress (Domínguez-Cuevas et al. 2006). Benzoate toxicity could cause the adoption of a similar energy-saving strategy by *P. putida* F1 and induce the apparent increase of chemotactic proteins in citrate-grown cells (i.e. the relative decrease of the specific proteins in benzoate).

### Comparison of *P. putida* shotgun proteomics studies on the degradation of monoaromatic hydrocarbons

In a previous study, Yun et al*.* (2011) investigated the global proteomic response of benzoate- versus succinate-grown cells of *Pseudomonas putida* KT2440. Following iTRAQ-labeling and LC-MS/MS analysis on an LTQ mass spectrometer, 570 proteins were identified and 107 of them were regarded as differentially expressed using less stringent criteria (e.g. fold-change > 1.3, no limitation for *p*-value). In line with our findings, Yun et al. (2011) observed the upregulation of several benzoate-degrading proteins and amino acid ABC transporters in benzoate-grown cells. In contrast, an opposite trend was reported for ribosomal and chemotaxis-related proteins, which were found to be down- and up-regulated, respectively, in benzoate cultures. Though, a quite limited interpretation was provided for those findings. It is also interesting that *P. putida* KT2440 showed a higher number of heat-shock and universal stress proteins when grown on succinate rather than on benzoate (six versus two proteins). This observation was contradictory to our results, as *P. putida* F1 exhibited an upregulation of five stress-proteins when benzoate was used as carbon source.

In another study, the proteomic response of *P. putida* P8 during its growth on benzoate and succinate was investigated using gel-based quantitative proteomics (Cao and Loh 2008). Besides the upregulation of eight proteins involved in benzoate degradation via catechol ortho- and meta-cleavage pathways, five chaperones and detoxification/stress proteins were upregulated in benzoate-grown *P. putida* P8. Similarly with our results, Cao and Loh (2008) suggested that the oxidative stress could be more severe during biodegradation of aromatic pollutants and it may cause the overexpression of stress proteins for the protection of cells against oxidative damage. In addition, the upregulation of seven dehydrogenase/reductase proteins was reported for *P. putida* P8 cells grown on benzoate, implying the existence of a redox balancing mechanism, similar to that observed for *P. putida* F1.

In a more recent study, Kasahara et al. (2012) used a label-free method to perform a semi-quantitative proteomic analysis of *P. putida* F1 and to investigate the degradation of benzene and other monoaromatic compounds. With the exception of Pput_2549, benzene degradation induced the upregulation of all the enzymes involved in catechol ortho-cleavage pathway. In contrast to our findings, the upregulation of several enzymes participating in the catechol meta-cleavage pathway was also observed. Besides pollutant catabolism, a detailed interpretation of proteomic data regarding other cellular processes was not available in that study for further comparison.

Our goal in the present work was to build a bridge between traditional physiological study and contemporary proteomic research. The former approach simplifies microbial growth by using a few readily available parameters and pseudo-stoichiometric variables (brutto-formula of cell mass, yield, SGR, etc.) to generate an integrated view of a complex cellular system. The proteomic approach uncovers the stoichiometry of known metabolic processes emanating from complete microbial genome sequencing data.

The physiological and proteomic approaches complement each other, and their combination can lead to a better understanding of bacterial response under different growth conditions. In particular, the comparative physiological study of *P. putida* growth on citrate and benzoate, followed by proteomic analysis, revealed unexpectedly diverse cellular mechanisms required for changing from one C-source to another. First, we confirmed earlier findings and detected all enzymes of the peripheral catabolism of benzoate through the catechol ortho-cleavage pathway. Two other upregulated catabolic enzymes suggested that the protocatechuate ortho-cleavage pathway could also play a role in benzoate degradation. Moreover, catabolic enzymes for benzoate were presumed to induce the observed upregulation of an arylsulfatase, which could be essential for the catabolism of sulfated aromatic hydrocarbons co-existing in nature with benzoate. A second category of proteins was upregulated to optimize uptake of respective exogenous C-substrates (i.e. specific benzoate and citrate transporters).

Finally, several other groups of proteins were found to be responsible for various physiological adjustments made by *P. putida* F1 in response to the C-source change. By examining the functions of these proteins, we were able to discern important information about altered cellular processes that otherwise would be difficult to deduce by a simple examination of respective metabolic pathways. Examples included: i) the induction of stress response (including the increase of ribosomal proteins as a side effect) resulting from the toxicity of benzoate; ii) the activation of redox-balancing due to the higher C:O ratio (degree of reduction) of benzoate versus citrate; iii) the induction of metabolic pH-regulation caused by a higher intracellular alkalization from trisodium citrate; and iv) the increase of cellular motility and chemosensory mechanisms in citrate relative to benzoate-grown cells as a result of benzoate toxic-effect. The results of the present study thus demonstrate the power of proteomics in the understanding of bacterial response to various C-sources including organic pollutants. This understanding should ultimately impact environmental engineering as the molecular processes operating with the bacterial cells become understood.

## Competing interests

The authors declare that they have no competing interests.

## Supplementary Material

Additional file 1 Electronic supplementary material. Click here for file
