# Supplementary material for: Comparative proteomic analysis reveals mechanistic insights into Pseudomonas putida F1 growth on benzoate and citrate
Source: AMB Express. 2013 Oct 25;3:64. doi: 10.1186/2191-0855-3-64 (PMC3827995; doi:10.1186/2191-0855-3-64)
Supplement: Additional file 1 — Electronic supplementary material. [file 2191-0855-3-64-S1.doc]

**Electronic supplementary material**

**Comparative proteomic analysis reveals mechanistic insights into Pseudomonas putida F1 growth on benzoate and citrate**

Manolis Mandalakis,^†,‡^ Nicolai Panikov,^†,§^ Shujia Dai,^†^ Somak Ray,^†^ Barry L. Karger^*,†^

† Barnett Institute, Northeastern University, Boston, MA 02115

‡ Present Address: Department of Chemistry, University of Crete, Heraklion, Greece, GR-71409

§ Present Address: Harvard School of Public Health, Department of Immunology and Infectious Diseases, Boston, MA 02115

* To whom correspondence should be addressed. B.L. Karger: E-mail: [b.karger@neu.edu](mailto:b.karger@neu.edu), Phone: 617.373.2867, FAX: 617.373.8795

**Computational analysis of shotgun proteomics data**

The proteomic data were processed using Proteome Discoverer (version 1.3; Thermo Fisher Scientific, Waltham, MA) and a local MASCOT server (version 2.3; Matrix Science, London, UK). The MS/MS spectra were searched against the protein database of *P. putida* F1, consisting of SwissProt and its TrEMBL supplement (5,245 protein entries by June 2012; http://www.uniprot.org/). A precursor ion tolerance of 10 ppm and a fragment ion tolerance of 0.8 Da (CID) or 0.05 Da (HCD) were specified. Trypsin with a maximum of two missed cleavages was selected; carbamidomethylation of cysteine, TMT labeling on both lysine and N-terminal amine of peptides were set as fixed modifications. Finally, a target-decoy approach (Elias and Gygi 2007) was applied to maintain the false discovery rate (FDR) below 1% for peptide identification. Proteins with at least two unique peptides were regarded as confident identifications, while all single-peptide hits were excluded from further analysis.

Relative quantitation of proteins was achieved by pairwise comparison of TMT reporter ion intensities among samples using DanteR software (version 0.1.1; Pacific Northwest National Laboratory, Richland, WA; http://omics.pnl.gov). Protein ratios were calculated as the geometric mean of the respective peptide ratios with their statistical significance assessed by ANOVA (Roxas and Li 2008). Peptides showing co-isolation interference higher than 25% were discarded from this analysis, and quantitation was applied only for proteins showing at least two peptide ratios. Furthermore, an empirical Bayesian analysis (EBA) reported by Margolin et al. (2009) and Wojcechowskyj et al. (2011) was applied to derive differentially expressed proteins. In essence, the log-ratio distribution of quantified proteins was modeled by a mixture of three independent component distributions. The central part of the data or the “null model” with no differential expression was assumed to be a Gaussian distribution, while the positive and negative tails, which correspond to the differentially expressed proteins, were modeled by a Generalized Pareto distribution. The parameters describing the distributions were derived by applying the method of maximum likelihood fitting, and the resulting values were used to estimate the complete marginal density of the distribution. The Bayesian posterior probability for each protein not belonging to the “null model” was estimated from the null and marginal density.

Based on the empirical Bayesian framework, the posterior probability of a protein showing differential abundance was defined by a local false discovery rate (FDR) threshold. The local FDR for threshold K is the sum of all the null probabilities for ratios that would be denoted as discoveries (i.e. that have a posterior probability of being non-null that is higher than K). By using EBA, the thresholds of ratios for differentially expressed proteins were set at a corresponding local FDR of 5% (i.e. fold-change < 0.68 or > 1.47). An additional threshold for the ANOVA p-values was set at <0.01 to keep only those proteins showing statistically significant changes.

The lists of up- and down-regulated proteins were divided into functional categories by performing an extensive search of several on-line protein function databases (KEGG Pathway Database, InterPro, UniProt). Protein lists were also subjected to Gene Ontology (GO) enrichment analysis using DAVID functional classification tool (Database for Annotation, Visualization and Integrated Discovery, http://david.abcc.ncifcrf.gov/) (Huang et al. 2009a, 2009b).

***References***

Elias JE, Gygi SP (2007) Target-decoy search strategy for increased confidence in large-scale protein identifications by mass spectrometry. Nat Methods 4:207–214

Roxas BA, Li Q (2008) Significance analysis of microarray for relative quantitation of LC/MS data in proteomics. BMC Bioinformatics 9:187

Margolin AA, Ong SE, Schenone M, Gould R, Schreiber SL, Carr SA, Golub TR (2009) Empirical Bayes analysis of quantitative proteomics experiments. PLoS ONE 4:e7454

Wojcechowskyj JA, Lee JY, Seeholzer SH, Doms RW (2011) Quantitative phosphoproteomics of CXCL12 (SDF-1) signaling. PLoS ONE 6:e24918

Huang DW, Sherman BT, Lempicki RA (2009a) Systematic and integrative analysis of large gene lists using DAVID bioinformatics resources. Nat Protoc 4:44–57

Huang DW, Sherman BT, Lempicki RA (2009b) Bioinformatics enrichment tools: paths toward the comprehensive functional analysis of large gene lists. Nucleic Acids Res 37:1–13

**Table S1.** List of preferentially expressed proteins in benzoate-grown cells.

**Table S1.** (continued)

**Table S2.** List of preferentially expressed proteins in citrate-grown cells.

**Table S2.** (continued)

**Table S3.** Kinetic model describing bacterial growth in the activated and balanced batch culture

| **State variable** | **Monod model** | | **Simplified Monod model** | |
| --- | --- | --- | --- | --- |
| Cell mass, *x* | $\frac{dx}{dt}=(\mu-a)x, \mu=\mu_{m}\frac{s}{K_{s}+s}$ | [1] | $\frac{dx}{dt}=\left\{\begin{matrix} \mu_{m}x if s>0 growth\text{ }phase \\ -ax if s=0 starvation \end{matrix}\right.$ | [1a] |
| Concentration of the C-source, *s* | $\frac{ds}{dt}=-\frac{\mu x}{Y}$ | [2] | $\frac{ds}{dt}=\left\{\begin{matrix} -\mu_{m}x/Y & if s>0 \\ 0 & if s=0 \end{matrix}\right.$ | [2a] |
| Respiratory CO_2_ (*p*) production | $\frac{dp}{dt}=Y_{p/x}\mu x+ax$ | [3] | $\frac{dp}{dt}=\left\{\begin{matrix} Y_{p/x}m_{m}x/Y & if s>0 \\ ax & if s=0 \end{matrix}\right.$ | [3a] |
| Dissolved oxygen (*O*) | $\frac{dO}{dt}=K_{L}(O_{0}-O)-Y_{o/x}(\mu x+ax)$ | [4] | $\frac{dO}{dt}=K_{L}(O_{0}-O)-\left\{\begin{matrix} -Y_{o/x}\mu_{m}x & if s>0 \\ -Y_{o/x}ax & if s=0 \end{matrix}\right.$ | [4a] |
| **Symbols**: *μ* specific growth rate, SGR; *μ_m_*, maximal SGR achieved under unrestricted growth conditions; *a,* specific turnover/death rate (could be considered as self-digestion of cellular polymers accounting maintenance requirements); *Y, Y_p/x_* and *Y_o/x_* are stoichiometric coefficients quantifying respectively cell mass yield per unit mass of consumed C-substrate, CO_2_ yield per mass unit of biomass and oxygen needed to produce unit of biomass; *K_L_*, oxygen mass-transfer rate between gas and liquid phases | | | | |

**Figure S1.** Volcano plot illustrating the fold-change versus p-values of *P. putida* F1 proteins expressed in benzoate or citrate grown cells. (Fold change is the ratio of protein levels in the benzoate versus citrate-grown cells). The horizontal dotted lines represent the p-value cut-off thresholds (p-value <0.01), while the vertical dotted lines represent the fold-change cut-off thresholds (fold change >1.47) derived from the empirical Bayesian analysis.

**Figure S2.** HPLC chromatograms (at 275 nm) of *P. putida* F1 culture supernatants over the course of benzoate degradation. The gradual accumulation of *cis,cis*-muconate (Peak 1) and catechol (Peak 2) intermediates with decreasing levels of benzoate are shown.
